# Maintenance of the bladder cancer precursor urothelial hyperplasia requires FOXA1 and persistent expression of oncogenic *HRAS*

**DOI:** 10.1038/s41598-018-36720-6

**Published:** 2019-01-22

**Authors:** Christopher H. Yee, Zongyu Zheng, Lauren Shuman, Hironobu Yamashita, Joshua I. Warrick, Xue-Ru Wu, Jay D. Raman, David J. DeGraff

**Affiliations:** 1 Department of Pathology and Laboratory Medicine, The Pennsylvania State University, College of Medicine, Hershey, PA USA; 2 Departments of Urology and Pathology, New York University School of Medicine and Veterans Affairs New York Harbor Healthcare System, Manhattan Campus, New York, NY USA; 3 Department of Surgery, Division of Urology, The Pennsylvania State University, College of Medicine, Hershey, PA USA; 4 Department of Biochemistry and Molecular Biology, The Pennsylvania State University, College of Medicine, Hershey, PA USA

## Abstract

Tumorigenesis requires accumulation of genetic and epigenetic alterations, some of which drive tumor initiation. “Oncogene addiction” describes the phenomenon that (1) well-established cancers are dependent on one mutated oncogene or pathway for the maintenance of a malignant phenotype and that (2) withdrawal of the single oncogenic event leads to growth arrest and/or cancer regression. While oncogene addiction has been experimentally validated in advanced tumor models, its role in tumor precursors has not been investigated. We utilized the requirement of Forkhead box A1 (*Foxa1*) for transcriptional activation of the *Upk2-*promoter to temporally control the expression of Upk2-HRAS* oncogene, an inducer of urothelial hyperplasia in transgenic mice. Inducible homozygous knockout of *Foxa1* in Upk2-HRAS*/UBC-Cre^*ERT2*^*/Foxa1*^*loxp/loxp*^ mice results in reduced HRAS* levels. This led to a marked reduction of urothelial proliferation as evidenced by urothelial thinning, degenerative changes such as intracellular vacuole formation, and reduced Ki67 expression. Reduced proliferation did not affect basal, Krt14-positive cells, supporting the fact that Foxa1-regulated *Upk2*-HRAS* expression occurs primarily in supra-basal cells. Our results indicate that maintenance of urothelial hyperplasia in Upk2-HRAS* mice depends on continuous expression of Foxa1 and activated HRAS, and that mutated receptor tyrosine kinases, FOXA1 and/or other downstream effectors may mediate oncogene addiction in urothelial hyperplasia.

## Introduction

Bladder cancer is the sixth most common cancer in the United States, with over 80,000 new diagnoses and 17,000 deaths estimated to occur in 2018^1^. Bladder cancer presents as two relatively distinct clinical entities—non-invasive and invasive disease—and these entities arise through largely independent molecular changes^2^. Approximately 70% of all newly diagnosed bladder cancers are noninvasive and are believed to progress from a state of urothelial hyperplasia. Urothelial hyperplasia and early stage, noninvasive bladder cancer frequently exhibit mutations in the gene encoding fibroblast growth factor receptor 3 (FGFR3) as well as components of the phosphoinositide 2-kinase/AKT/mammalian target of rapamycin (PI3K/AKT/mTOR) pathway^3^.

The mechanism(s) through which develolpment of urothelial hyperplasia, tumorigenesis, and progression to frank carcinoma occurs following alterations in FGFR3 and PI3K/AKT/mTOR are complex and multifactorial. For example, studies show activating mutations in Fgfr3 (K644E) alone have minimal impact on urothelial proliferation *in vivo*, but they appear to cooperate with knockout (KO) of the negative regulator of PI3K/AKT/mTOR signaling, phosphatase and tensin homologue (Pten) to drive urothelial hyperplasia and abnormal urothelial differentiation^4,5^. In addition, *Pten* KO has been associated with the development of urothelial hyperplasia and development of noninvasive bladder cancer in a subset of mice^6^, suggesting a major role for the PI3K/AKT/mTOR pathway and potentially other downstream pathway components. HRAS lies downstream of FGFR3, and increased signaling following mutations in HRAS are correlated with enhanced susceptibility to bladder cancer and other abnormalities in patients with Costello syndrome^7–12^. Indeed, creation of the Upk2-HRAS* model of bladder cancer confirmed a role for mutant HRAS in noninvasive bladder cancer^13^. In this model, expression of one copy of mutant HRAS results in simple urothelial hyperplasia, while expression of two copies of mutant HRAS results in noninvasive disease, complete with papillary structures. However, although mutation of HRAS as well as FGFR3 and PI3K/AKT/mTOR components are associated with development of noninvasive bladder cancer, it is unknown if the hyperplastic and/or transformed state depends upon maintenance of oncogene expression. This is important because confirmation of “oncogene addiction” (reviewed in^14,15^) to mutant HRAS or interrelated components of the FGFR3 or PI3K/AKT/mTOR pathways would allow development of targeted approaches to treat noninvasive bladder cancer.

To test the dependency of urothelial hyperplasia on HRAS* expression, we took advantage of previous observations indicating a direct role for forkhead box A1 (FOXA1) in the regulation of uroplakin 2 (*UPK2*). Specifically, we inducibly ablated *Foxa1* expression in the previously described Upk2-HRAS* model^13^ of urothelial hyperplasia. This approach enabled us to directly determine the impact of reduced Upk2 activity and associated oncogene expression, as well as *Foxa1* KO on urothelial hyperplasia. Our results demonstrate a role for continuous HRAS* and/or Foxa1 expression in the maintenance of urothelial hyperplasia.

## Materials and Methods

### Mouse lines for breeding and experiments

All animal studies were conducted in accordance with government guidelines and under an active protocol approved by the Pennsylvania State University College of Medicine Institutional Animal Care and Use Committee. Upk2-HRAS*^13^, *UBC-Cre*^*ERT2* ^^16^, and *Foxa1*^*loxp/loxp *^^17^ mice were previously described. At 3 months of age, genetic control mice, Upk2-HRAS*/UBC-Cre^*ERT2*^*/Foxa1*^*loxp*^ mice and Upk2-HRAS*/UBC-Cre^*ERT2*^*/Foxa1*^*loxp/loxp*^ mice were intraperitoneally injected with 1 mg/day of tamoxifen for 5 days and mice were euthanized 3 months after tamoxifen injection. In a subset of experiments (see supplemental data), mice bearing two copies of Upk2-HRAS* were bred with *Upk2-Cre/Foxa1*^*loxp/loxp*^ mice. *Upk2-Cre*^18^ mice have been previously described. Experimental and appropriate controls were euthanized at six months of age. Bladders were dissected, formalin fixed, processed, and paraffin embedded. The following primers (Eurofins, Lancaster, PA) were used for genotyping: Upk2-Cre-F: 5′-CGTACTGACGGTGGGAGAAT-3′, Upk2-Cre-R: 5′-TGCATGATCTCCGGTATTGA-3′, HaRas-F: 5′-TCCCACTCCGAGACAAAATC-3′, HaRas-R: 5′-ATTCGTCCACGAAGTGGTTC-3′, FoxA1-F: 5′-CTGTGGATTATGTTCCTGATC-3′, FoxA1-R: 5′-GTG TCAGGATGCCTATCTGGT-3′, UBC-F: 5′-GCGGTCTGGCAGTAAAAACTATC-3′ (oIMR1084, Jackson Laboratory, Cincinnati, OH), and UBC-R: 5′-GTGAAACAGCATTGCTGTCACTT-3′ (oIMR1085, Jackson Laboratory).

### Immunohistochemistry (IHC)

Freshly dissected tissue was fixed in PBS-buffered 10% formalin and processed by routine methods prior to paraffin embedding. Sections (5 μm) were stained with hematoxylin and eosin (H&E) as previously reported^19^. Urothelial thickness was measured by CellSens Entry (Olympus America Inc, Center Valley, PA). IHC was performed as previously described^18^. Briefly, slides were deparaffinized and rehydrated through a series of graded alcohols and washed in deionized water for 3 minutes. Antigen retrieval was performed by placing slides in 1% antigen unmasking solution (Vector Labs, Burlingame, CA) and heating slides for 20 minutes on high power in a pressure cooker (Cuisinart CPC-600, Conair Corporation, Stamford, CT). Steam was released in short bursts to prevent boiling and to preserve tissue integrity. Slides were cooled to room temperature and washed for three 10-minute washes in phosphate-buffered saline (PBS; pH 7.4). All incubations were performed at room temperature unless otherwise noted. Endogenous peroxidases were blocked by incubation in 1% hydrogen peroxide in methanol for 20 minutes, and slides were again washed for three 10-minute washes in PBS. Sections were incubated in PBS containing horse serum (Vector Labs) for 1 hour to reduce nonspecific antibody binding and then incubated overnight with primary antibody at 4 °C in a humidified chamber. Primary antibodies used for IHC include goat anti-Foxa1 (1:1000; Santa Cruz Biotechnology, Santa Cruz, CA), rabbit anti-Gata3 (1:1000; Sigma-Aldrich, St. Louis, MO), mouse anti-cytokeratin 14 (Krt14; 1:200; Vector Labs), rabbit anti-Ki67 (1:1000; Abcam, Cambridge, UK) and goat anti-Fabp4 (1:200, R&D Systems, Minneapolis, MN). Following overnight incubation, slides were washed in three 10-minute PBS washes, and sections were incubated in biotinylated secondary antibody diluted in PBS containing horse serum (1:200; Vector Labs) for 1 hour. Specific antibody binding was visualized using Vectastain Elite ABC Peroxidase kit (Vector Labs) according to the manufacturer protocol with diaminobenzidine substrate buffer as the chromogen (Thermo Fisher Scientific, Waltham, MA). Immunohistochemistry for Ki67 was scored by calculating the average number of positive cells per 100 cells in 4 high powered fields. Statistically significant differences in Ki67 immunopositivity were identified via the application of Kruskal-Wallis H test. P values are reported following application of Dunn’s test to correct for multiple comparisons.

### Western blotting

All bladder tissues were homogenized in Trizol (Thermo Fisher Scientific) per manufacturer instructions, and protein was extracted following isolation of RNA and DNA. Protein concentrations were measured using the Pierce BCA Protein Assay Kit (Thermo Fisher Scientific) as per manufacturer’s instructions. Following extraction, protein samples (40 μg of, 1x LDS sample buffer, 10% 2-mercaptoethanol) were electrophoresed on 4–12% Bis-Tris NuPAGE gels (Thermo Fisher Scientific), and proteins were subsequently transferred to nitrocellulose blotting membrane (GE Healthcare Life Science, Pittsburgh PA) using a Pierce G2 Fast Blotter (Thermo Fisher Scientific) according to manufacturer protocol. Following transfer, membranes were incubated at room temperature in 5% non-fat milk (NFDM) dissolved in Tris buffered saline containing 0.1% Tween-20 (TBST) for 1 hour. Additionally, all primary antibodies used in this study were diluted in TBST with 5% NFDM. Dilutions of primary antibodies were as follows: anti-Foxa1 (ab23738, Abcam), anti-HRAS (PA5-22392, Thermo Fisher Scientific), anti-E-Cadherin (3169; Cell Signaling Technology, Danvers, MA), anti-pan-cytokeratin (ab27988, Abcam), and anti-Gapdh (14C10; Cell Signaling Technology). After incubation with primary antibodies overnight at 4 degrees C, all membranes underwent five 5-minute TBST washes. Secondary antibody (ECL anti-rabbit or mouse IgG, HRP-linked whole antibody (GE Healthcare Life Science) was diluted in TBST containing 5% NFDM and incubated at room temperature for one hour. After incubation with secondary antibodies, membranes underwent five 5-minute TBST washes. Protein bands were visualized by exposing membrane after addition of ECL Western Blotting Substrate (Thermo Fisher Scientific) to X-ray film (Thermo Fisher Scientific) via standard procedures.

RNA Extraction, Reverse Transcription, and Quantitative Real Time PCR (Q-RT-PCR). Dissected tissue was placed in RNAlater (Thermo Fisher Scientific) to stabilize RNA, and RNA was extracted using Trizol (Thermo Fisher Scientific) as per manufacturer protocol. Reverse transcription was conducted for 1 ug RNA/per sample using SuperScriptII (Thermo Fisher Scientific). Q-RT-PCR was performed using QuantStudio7 Real-Time PCR System using a 96 well format. Custom Taqman probes were designed for HRas and Gapdh detection. The sequences for the probe and primer sets were as follows: HRas-F: 5′-CCGGCGGTGTAGGCAAGAG-3′, HRas-R: 5′-TCGTCCACGAAGTGGTTCTG-3′, HRas-Probe: 6FAMGCACTGACCATCCAGCMGBNFQ, GAPDH-F: 5′-GGCAAATTCAACGGCACAGT-3′, GAPDH-R: 5′-CGCTCCTGGAAGATGGTGAT-3′, GAPDH-Probe: VICAAGGCCGAGAATGGMGBNFQ. Reactions consisted of 4 ul of cDNA, 10 μl of 2 × Taqman Gene Expression Master Mix, 0.5 uL of 1:10 dilutions of each primer and probe, and nuclease-free water to a total reaction volume of 20 μl/well. Relative gene expression was analyzed using the ΔΔCt method using Gapdh as a reference.

## Results

### Upk2-HRAS* bladders exhibit decreased mutant HRAS expression following inducible *Foxa1* KO

**Figure 1:**
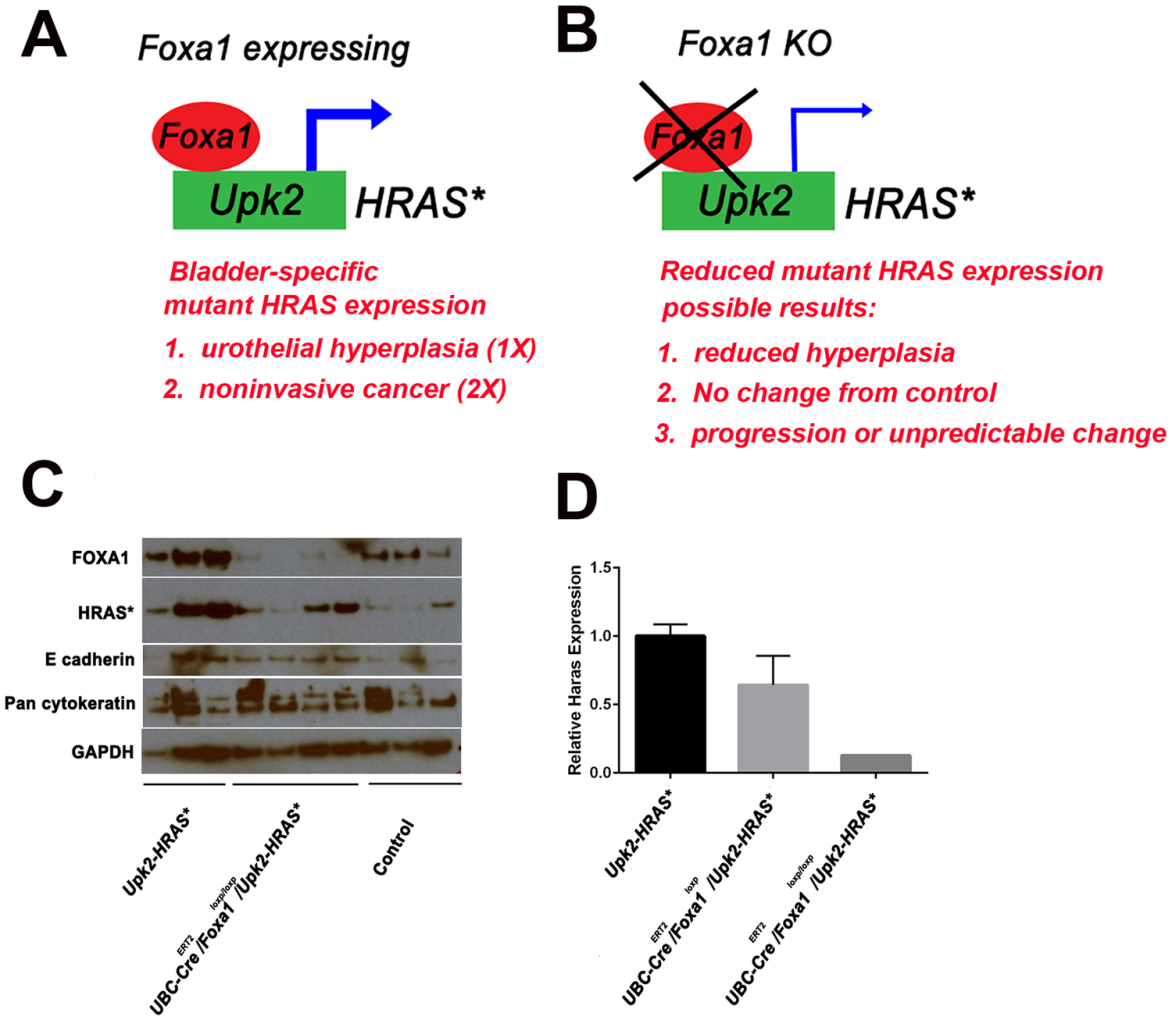
(**A**) Expression of one copy of mutant HRAS driven by the *Upk2* promoter results in urothelial hyperplasia, while expression of two copies of mutant HRAS results in noninvasive bladder cancer (NIBC). However, because FOXA1 is a direct regulator of UPK2 in human cells^20^, we hypothesized that *Foxa1* KO would (**B**) result in one of three possibilities: (1) decreased *Upk* promoter activity, decreased mutant HRAS expression, and regression of urothelial hyperplasia in Upk2-HRAS* mice; (2) increased proliferation and/or squamous differentiation in Upk2-HRAS* mice; or no impact/unpredictable results. (**C**) Western blotting of protein lysates for HRAS*, Foxa1, E-cadherin and pan-cytokeratin following bladder dissection. (C) Q-RT-PCR using probes specific for HRAS* expression. These results indicate that *Foxa1* KO results in reduced expression of HRAS* in the Upk2-HRAS* mouse model.

We took advantage of previous reports indicating FOXA1 was a direct positive regulator of *UPK2* expression^20^, and we devised a system to test the impact of reduced mutant HRAS expression following Foxa1 knockout in the Upk2-HRAS* mice on urothelial hyperplasia (Fig. 1A,B). To determine if persistence of urothelial hyperplasia required maintenance of HRAS* and/or Foxa1 expression, we bred Upk2-HRAS* mice with *UBC-Cre*^*ERT2*^*/Foxa1*^*loxp/loxp*^ mice. Resultant Upk2-HRAS*/UBC-Cre^*ERT2*^*/Foxa1*^*loxp*^ and Upk2-HRAS*/UBC-Cre^*ERT2*^*/Foxa1*^*loxp/loxp*^ mice, as well as all control genotypes were then injected with tamoxifen for five days and then aged for an additional three months. As expected, western blot analysis revealed *Foxa1* KO was associated with decreased expression of mutant HRAS but was not associated with significant alterations in expression of the epithelial markers E cadherin or cytokeratins (Fig. 1C). Decreases in mutant HRAS expression following *Foxa1* KO were further confirmed by Q-RT-PCR (Fig. 1D). These observations strongly suggest that Foxa1 regulates *Upk2* promoter activity, thus resulting in decreased HRAS expression following *Foxa1* KO.

### HRAS*-induced hyperplasia regresses following inducible *Foxa1* KO

**Figure 2:**
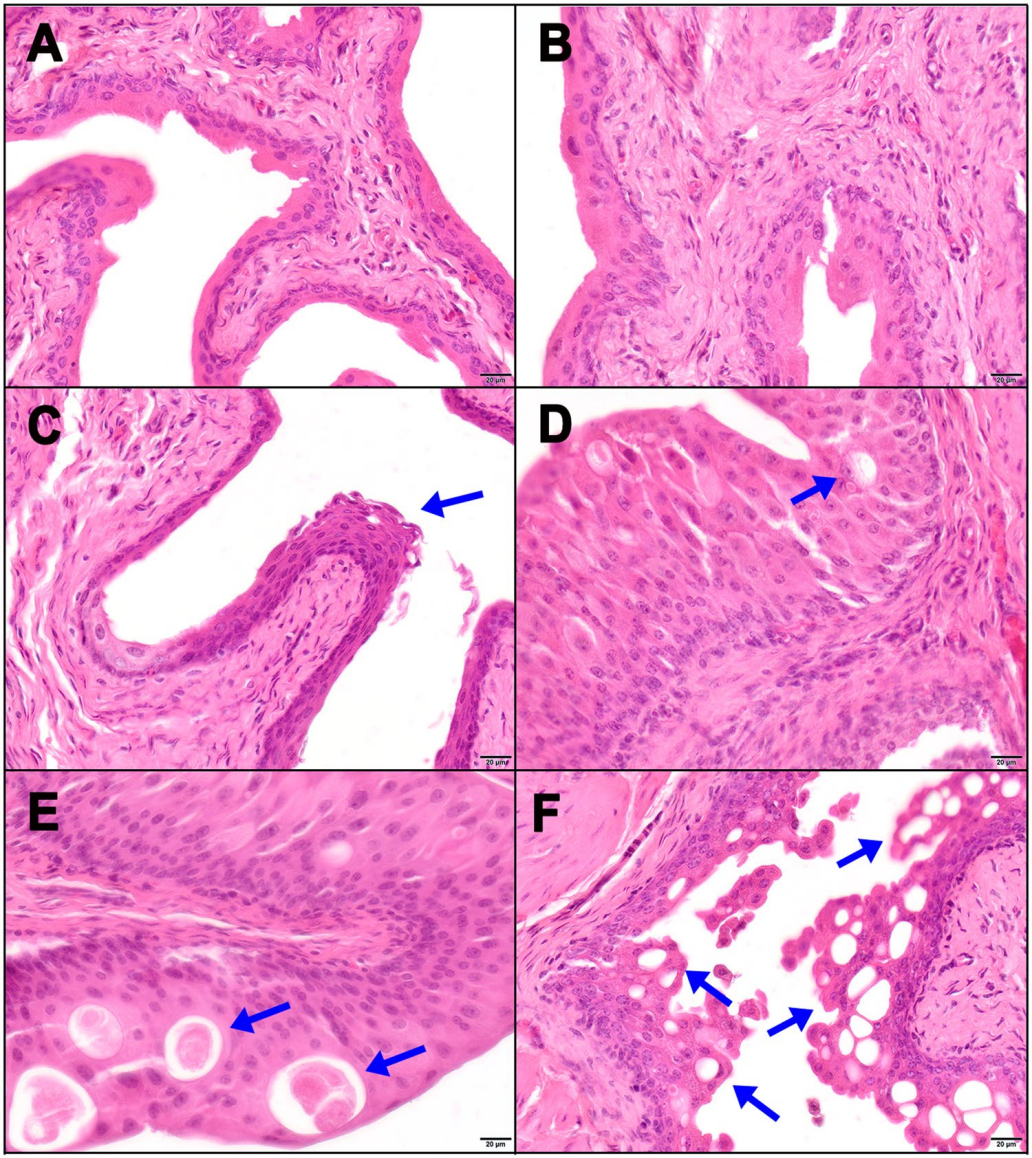
Foxa1 knockout in the setting of *Upk2*-driven expression of mutant HRAS results in regression of urothelial hyperplasia with a cystic phenotype. (**A**) Genetic control (*UBC-Cre*^*ERT2*^ alone) mice, (**B**) *UBC-Cre*^*ERT2*^*/Foxa1*^*loxp*^ and (**C**) *UBC-Cre*^*ERT2*^*/Foxa1*^*loxp/loxp*^ mice were injected with tamoxifen once daily for five days and then sacrificed 3 months later. Note presence of squamous metaplasia in UBC-Cre^ERT2^/Foxa1^loxp/loxp^ mice (panel c) as previously reported. All Upk2-HRAS* mice were similarly injected with tamoxifen and sacrificed 3 months later. (**D**) Upk2-HRAS* with minimal cysts, (**E**) Upk2-HRAS*/UBC-Cre^*ERT2*^*/Foxa1*^*loxp*^ mice with increased cyst presence, and (**F**) Upk2-HRAS*/UBC-Cre^*ERT2*^*/Foxa1*^*loxp/loxp*^ mice with extensive cyst formation and regression of urothelial hyperplasia.

**Figure 3:**
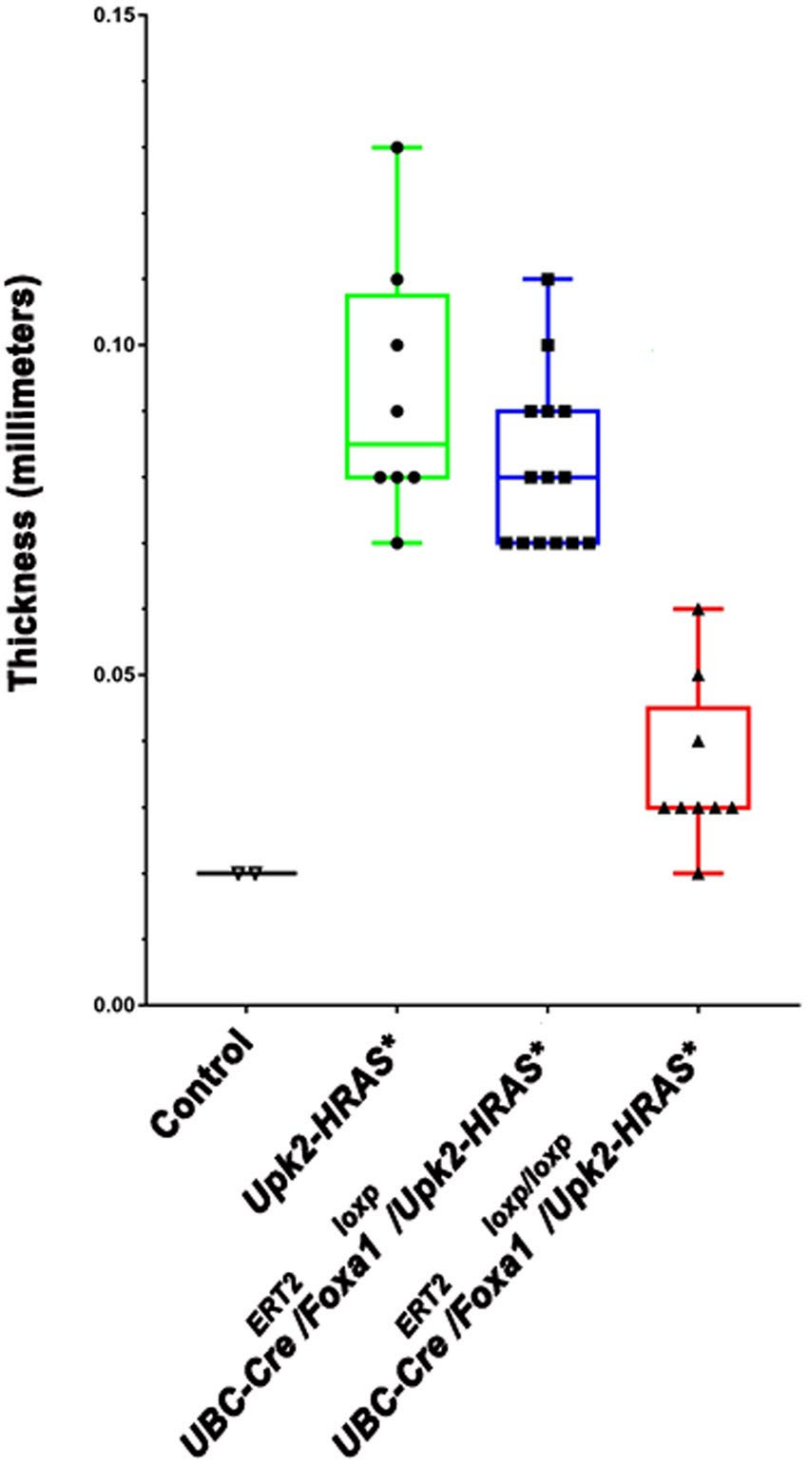
Urothelial thickness is significantly decreased following *Foxa1* knockout in Upk2-HRAS* mice. Urothelial thickness is significantly increased in Upk2-HRAS* mice relative to control (P = 0.006; Kruskal-Wallis H test), confirming the presence of urothelial hyperplasia. While KO of one copy of Foxa1 in Upk2-HRAS* mice has minimal impact compared to control (P = 0.03; Kruskal-Wallis H test), urothelial thickness following homozygous Foxa1 KO in Upk2-HRAS* mice was not significantly thicker than control urothelium.

Following confirmation of reduced HRAS* expression following *Foxa1* KO, we next examined urothelial morphologies associated with our mouse genotypes. Representative H&E-stained sections of bladder urothelium are pictured in Fig. 2. As previously reported, compared to urothelium from control mice (Fig. 2A), KO of one allele of *Foxa1* resulted in no detectable histologic changes in urothelium (Fig. 2B), while KO of two alleles resulted in the development of keratinizing squamous metaplasia^19^ (Fig. 2C). Also as previously reported, overexpression of one copy of mutant HRAS resulted in the development of urothelial hyperplasia^13^ (Fig. 2D). While KO of one allele of *Foxa1* in the setting of overexpressing one copy of mutant HRAS had no detectable effect (Fig. 2E), homozygous *Foxa1* KO in this setting dramatically decreased urothelial thickness (Fig. 2F) and resulted in additional structural changes. For example, while mutant HRAS-overexpressing urothelium demonstrated intraepithelial inclusions filled with eosinophilic material (Fig. 2D,E), urothelium of homozygous *Foxa1* KO animals demonstrated the widespread presence of vacuolar structures on H&E staining (Fig. 2F). In addition, significant urothelial thickening relative to control following overexpression of one copy of mutant HRAS (P = 0.0064; Kruskal-Wallis H test) was abolished following homozygous *Foxa1* KO (P = 0.99; Kruskal-Wallis H test) (Fig. 3). Similar results were observed through the use of an *Upk2-Cre* line to ablate *Foxa1*, even when two alleles of mutant HRAS were expressed (Supplementary Fig. 1). These observations indicate that inducible *Foxa1* KO abolishes hyperplasia directly and/or by decreasing expression of *Upk2-*driven mutant HRAS* following overexpression of one copy of mutant HRAS.

### Homozygous *Foxa1* KO results in reduced proliferation and Ki67 expression, but has no effect on the expression of luminal and basal markers

**Figure 4:**
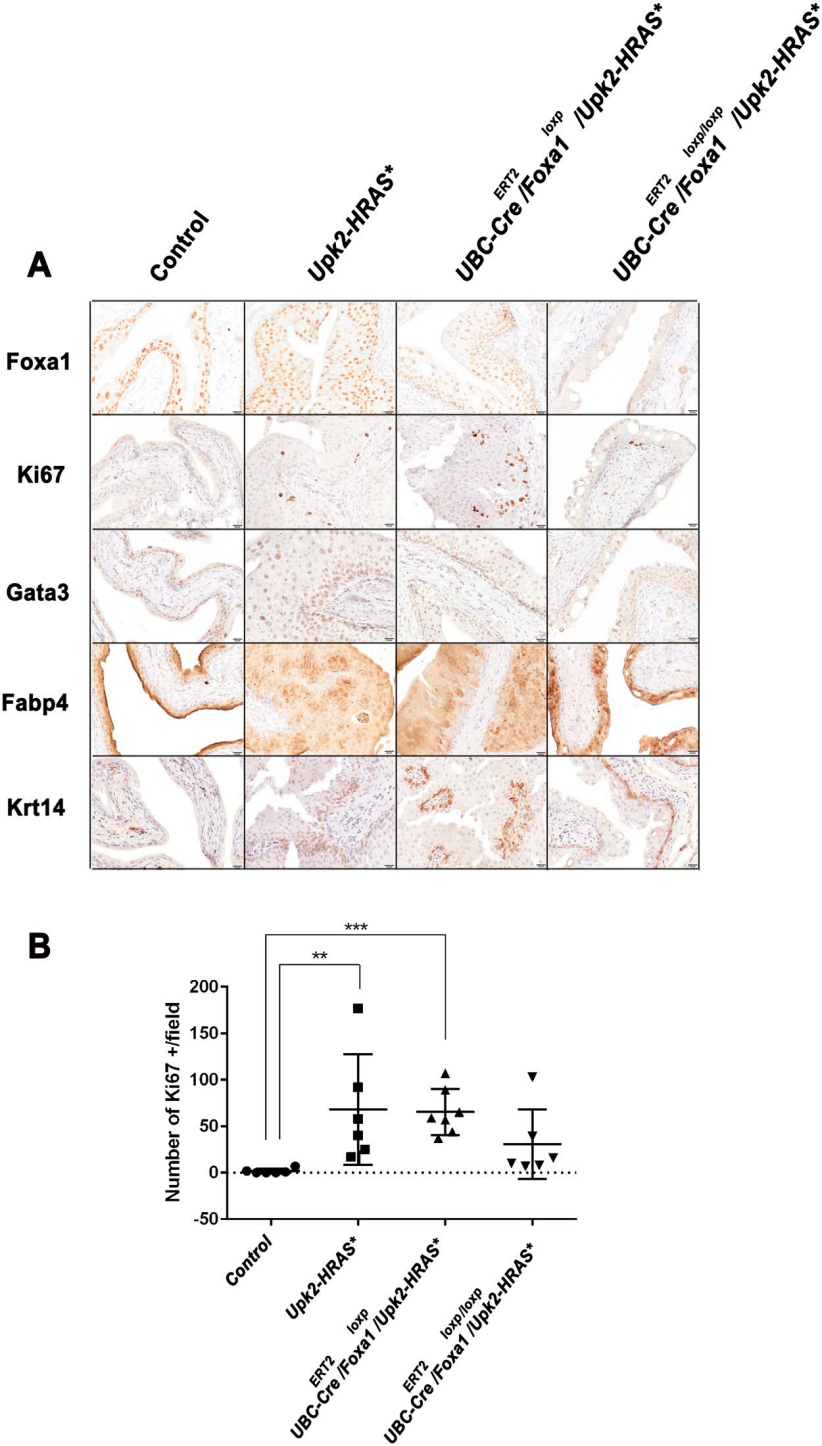
(**A**) Homozygous Foxa1 knockout reduces urothelial proliferation in Upk2-HRAS* mice but has no impact on global expression of luminal gene expression markers. Immunohistochemistry for Foxa1 confirms KO, while Ki67 staining reveals reduced proliferation following *Foxa1* homozygous KO. Conversely, heterozygous and homozygous Foxa1 KO had no impact on the staining patterns of the luminal markers Gata3 and or the basal marker Krt14. Fabp4 staining shifted from being predominantly nuclear and enriched in the umbrella cell population to being diffusely positive in HRAS* mutant mice with wild-type *Foxa1* or heterozygous *Foxa1* KO. Following *Foxa1* KO, Fabp4 was predominantly nuclear. (**B**) Quantification of Ki67 in control and following *UBC-Cre*^*ERT2*^ induced knockout of one and two alleles of *Foxa1*. Compared to control tissue, Ki67 expression was significantly higher in heterozygous Upk2-HRAS* mice (P = 0.004; Kruskal-Wallis H test), as well as in heterozygous Upk2-HRAS* following *UBC-Cre*^*ERT2*^*-*mediated deletion of one allele of *Foxa1* (P = 0.0007; Kruskal-Wallis H test). However, knockout of both alleles of *Foxa1* in Upk2-HRAS* mice resulted in the detection of Ki67 levels similar to control (P = 0.16). Therefore, *Foxa1* knockout results in reduced proliferation in the Upk2-HRAS* model of urothelial hyperplasia.

To confirm *Foxa1* KO, as well as to examine the association between morphologic changes and expression of markers of proliferation and gene expression subtype, we performed immunohistochemistry (IHC) on bladder specimens collected from control and experimental mice (Fig. 4). As expected, we failed to detect Foxa1 expression in bladder tissue dissected from Upk2-HRAS*/UBC-Cre^*ERT2*^*/Foxa1*^*loxp/loxp*^ mice. In addition, while detection of the proliferation marker Ki67 was significantly increased in Upk2-HRAS* (Fig. 4A,B; P = 0.004; Kruskal-Wallis H test) and Upk2-HRAS*/UBC-Cre^*ERT2*^*/Foxa1*^*loxp*^ (Fig. 4A,B; P = 0.0007; Kruskal-Wallis H test) mice compared to control, Ki67 expression in Upk2-HRAS*/UBC-Cre^*ERT2*^*/Foxa1*^*loxp/loxp*^ mice was not significantly different from control (Fig. 4A,B; P = 0.16; Kruskal-Wallis H test), which is consistent with reduced urothelial thickness following *Foxa1* KO. We previously reported that *Foxa1* KO induces squamous metaplasia^19^, and others have shown FOXA1 expression is significantly decreased in the basal-squamous gene expression subtype^21^, which is consistent with our previous findings^22^. Therefore, we performed IHC for the luminal markers Gata3 and Fabp4, as well as the basal-squamous marker Krt14. Our samples exhibited no differences in expression of the luminal markers Gata3 and Fabp4 or of the basal marker Krt14 between Upk2-HRAS*/UBC-Cre^*ERT2*^*/Foxa1*^*loxp*^ and Upk2-HRAS*/UBC-Cre^*ERT2*^*/Foxa1*^*loxp/loxp*^ mice (Fig. 4A). However, Fabp4 did exhibit a genotype-specific localization pattern. For example, control urothelium exhibited high cytoplasmic and nuclear Fabp4 expression in superficial umbrella cells, with nuclear expression in underlying urothelium. Upk2-HRAS* expressing urothelium conversely exhibited predominant membrane staining pattern for Fabp4 throughout the urothelium, with patchy areas of nuclear staining. *UBC-Cre*^*ERT2*^/Upk2-HRAS*/Foxa1^*loxp/loxp*^ mice exhibited a predominant nuclear staining pattern (Fig. 4A). However, these data suggest *Foxa1* KO does not impact global gene expression subtype following mutant HRAS-induced urothelial hyperplasia.

## Discussion

In the Upk2-HRAS* mouse model, expression of one copy of HRAS* results in urothelial hyperplasia, and our data indicate inducible KO of *Foxa1* results in reduced *Upk2* promoter activity, reduced HRAS* expression, and decreased urothelial proliferation. These findings suggest that maintenance of urothelial hyperplasia in the Upk2-HRAS* line depends on chronic HRAS* expression. This conclusion is supported by observations at the biochemical (Fig. 1) and morphologic (Figs 2 and 3) levels as well as by the observation that reduced HRAS* expression is associated with decreased Ki67 detection following *Foxa1* KO (Fig. 4).

In addition, although we previously reported that *Foxa1* KO using the *UBC-Cre*^*ERT2*^ system results in squamous metaplasia and urothelial hyperplasia in a subset of mice^19^ (Fig. 2), morphological analysis and IHC show that the urothelial/luminal gene expression pattern is preserved following *Foxa1* KO in Upk2-HRAS* mice (Fig. 2). It is not clear why these tissues retain a urothelial/luminal phenotype. These *in vivo* experiments were terminated three months after inducible Foxa1 KO, and it is possible that the likelihood of squamous differentiation would increase in experimental mice over time. While we failed to detect any differences in the absolute value of Gata3, Fabp4 or Krt14 expression, we did note differences in Ki67 staining and changes in the localization of Fabp4 (Fig. 4). In addition to being a target of Pparɣ, Fabp4 shuttles fatty acids (which serve as ligands for Pparɣ) into the nucleus. While the significance of differences in Fabp4 localization are unknown, this may indicate a change in cell metabolism following HRAS* expression and Foxa1 KO. Nonetheless, the retention of a urothelial/luminal gene expression pattern in combination with reduced Ki67 following *Foxa1* KO further suggests that the predominant influence of *Foxa1* KO is to reduce *Upk2* promoter activity in this system, thus resulting in decreased HRAS* expression and reduced urothelial thickness. Moreover, our findings agree with previous reports that FOXA1 may bind and directly regulate the uroplakin promoters, particularly that of human *UPK2*^20^.

An alternate interpretation for our findings is that decreases in urothelial hyperplasia following *Foxa1* KO are a direct result of ablating *Foxa1*, unrelated to decreased Upk2-HRAS* expression. Alternatively, *Foxa1* KO could cooperate with reduced HRAS* expression. Based on previous findings showing that *Foxa1* KO results in urothelial hyperplasia and squamous differentiation^19^, it seems paradoxical that *Foxa1* KO would result in urothelial atrophy/reduced hyperplasia in Upk2-HRAS* mice. However, the context within these experiments is not that of normal urothelium, so this is entirely possible.

Expression of two copies of mutant HRAS results in the development of noninvasive bladder cancer at 6 months, and we saw identical urothelial regression in these animals following *Upk2*-Cre mediated *Foxa1* KO (see supplemental data). The fact that *Upk2-Cre* reduced urothelial hyperplasia even in the setting of high expression (two copies) of Upk2-HRAS* suggests the phenotypic consequences of any resultant genomic instability^23^ are not sufficient to overcome *Foxa1* KO and/or addiction to activated HRAS expression. Still, *Foxa1* KO was induced relatively early (3 months of age in experiments using *UBC-Cre*^*ERT2*^, embryonic day 12 for *Upk2-Cre*) in all experiments, and perhaps *Foxa1* KO would fail to reduce urothelial hyperplasia/tumor burden following prolonged HRAS* expression because of enhanced genetic instability.

Weaknesses of this study include our inability to measure the impact of *Foxa1* KO on urothelial proliferation in real time, and the fact that we did not use an inducible system to reversibly control Upk2-HRAS* expression after the development of urothelial hyperplasia. The creation of additional experimental systems will enable us to determine the exact contribution of each of these factors to changes in urothelial thickness. However, these results suggest alterations in FGFR and downstream pathways—including those of HRAS and/or PI3K/AKT/mTOR—may mediate oncogene addiction and/or that Foxa1 expression is required to maintain tumor proliferation. These may thus be targetable pathways for the management of non-invasive bladder cancer. Indeed, a potential role for these factors in oncogene addiction is suggested by the fact that these pathways are commonly altered in early stage disease^3^.

## Electronic supplementary material

Supplemental data

## Data Availability

All data generated or analyzed during this study are included in this published article. All datasets generated during and/or analyzed during the current study are available from the corresponding author on reasonable request.
