# Supplementary material for: Maintenance of the bladder cancer precursor urothelial hyperplasia requires FOXA1 and persistent expression of oncogenic HRAS
Source: Sci Rep. 2019 Jan 22;9:270. doi: 10.1038/s41598-018-36720-6 (PMC6342925; doi:10.1038/s41598-018-36720-6)

Maintenance of the bladder cancer precursor urothelial hyperplasia requires FOXA1 and persistent expression of oncogenic *HRAS*

Christopher H. Yee[1,†], Zongyu Zheng[1,†], Lauren Shuman[1], Hironobu Yamashita[1], Joshua I. Warrick[1], Xue-Ru Wu[2], Jay D. Raman[3], David J. DeGraff[1,3,4,*]

[1]Department of Pathology and Laboratory Medicine, The Pennsylvania State University, College of Medicine, Hershey, PA
[2]Departments of Urology and Pathology, New York University School of Medicine and Veterans Affairs New York Harbor Healthcare System, Manhattan Campus, New York, NY
[3]Department of Surgery, Division of Urology, The Pennsylvania State University, College of Medicine, Hershey, PA
[4]Department of Biochemistry and Molecular Biology, The Pennsylvania State University, College of Medicine, Hershey, PA
[†]These authors contributed equally

Supplemental Data

Supplemental Figure 1: Impact of Upk2-Cre induced *Foxa1* KO on urothelial thickness in homozygous HRAS* mice. Bladder tissue dissected from 6 month old mice expressing two copies of mutant HRAS (*Upk2-HRAS***) and (**A**) wild-type *Foxa1* (*Upk2-Cre/Upk2-HRAS**),* (**B**) heterozygous knockout of *Foxa1*(*Upk2-Cre/Upk2-HRAS**/Foxa1*^{loxp}); and (**C**) homozygous knockout of *Foxa1* (*Upk2-Cre/Upk2-HRAS**/Foxa1*$^{loxp/loxp}$). (**D**) Shows quantification of urothelial thickness in control and experimental mice. While urothelium measured in mice expressing two copies of *Upk2-HRAS** alone and in combination with heterozygous knockout of *Foxa1* was significantly thicker than control tissue (p=0.0004 and p=0.02; Kruskal-Wallis H test; Dunn's test for multiple comparisons), homozygous *Foxa1* knockout resulted in urothelium that was not significantly thicker than control mice (P = 0.45; Kruskal-Wallis H test, Dunn's test for multiple comparisons).

Supplemental Figure 2: Films from HRAS and FOXA1 Western blotting.

Supplemental Figure 3: Films from Pan Cytokeratin Western blotting.

Supplemental Figure 4: Films from E-Cadherin Western blotting.

Supplemental Figure 1

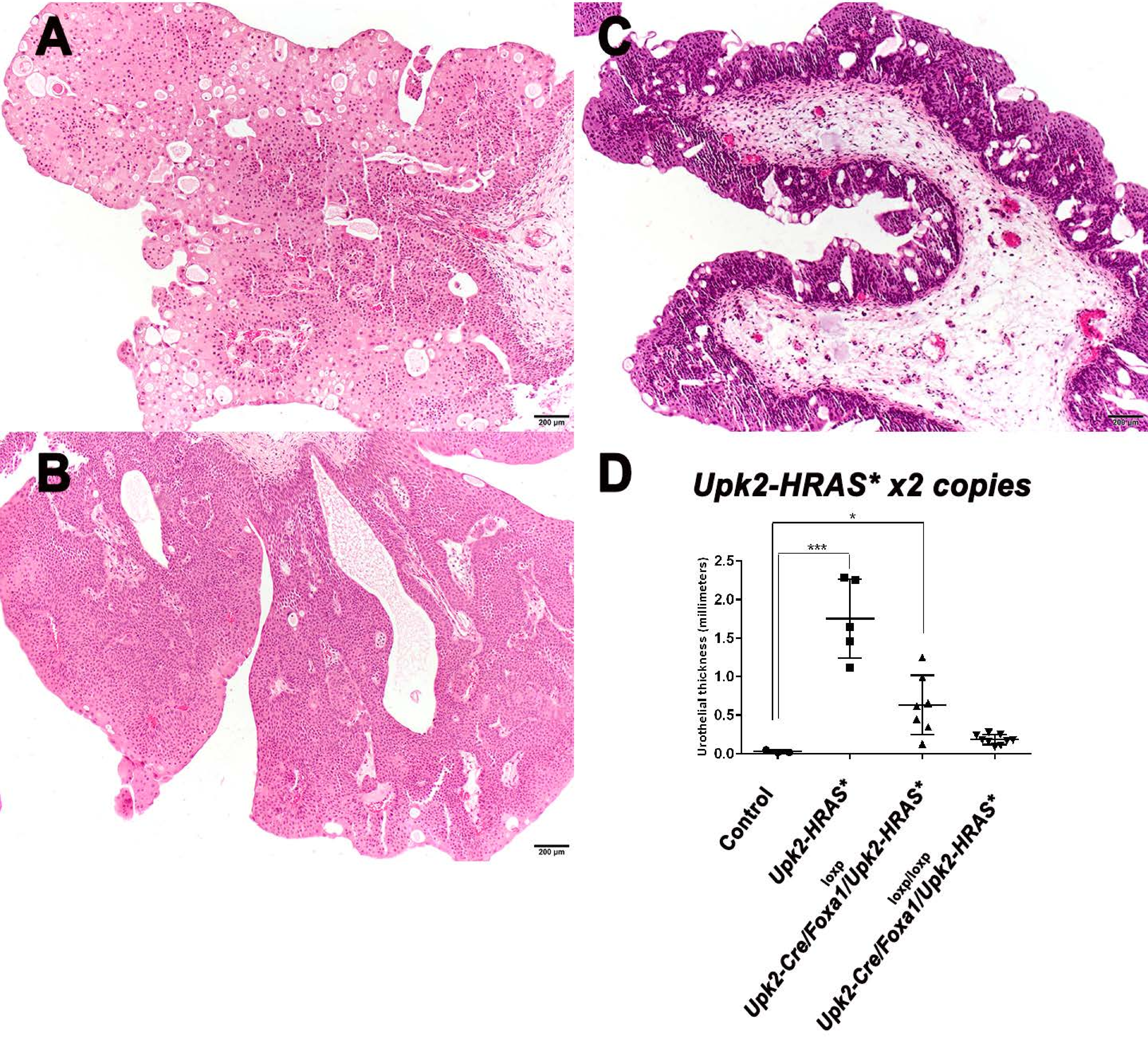

Supplemental Figure 2

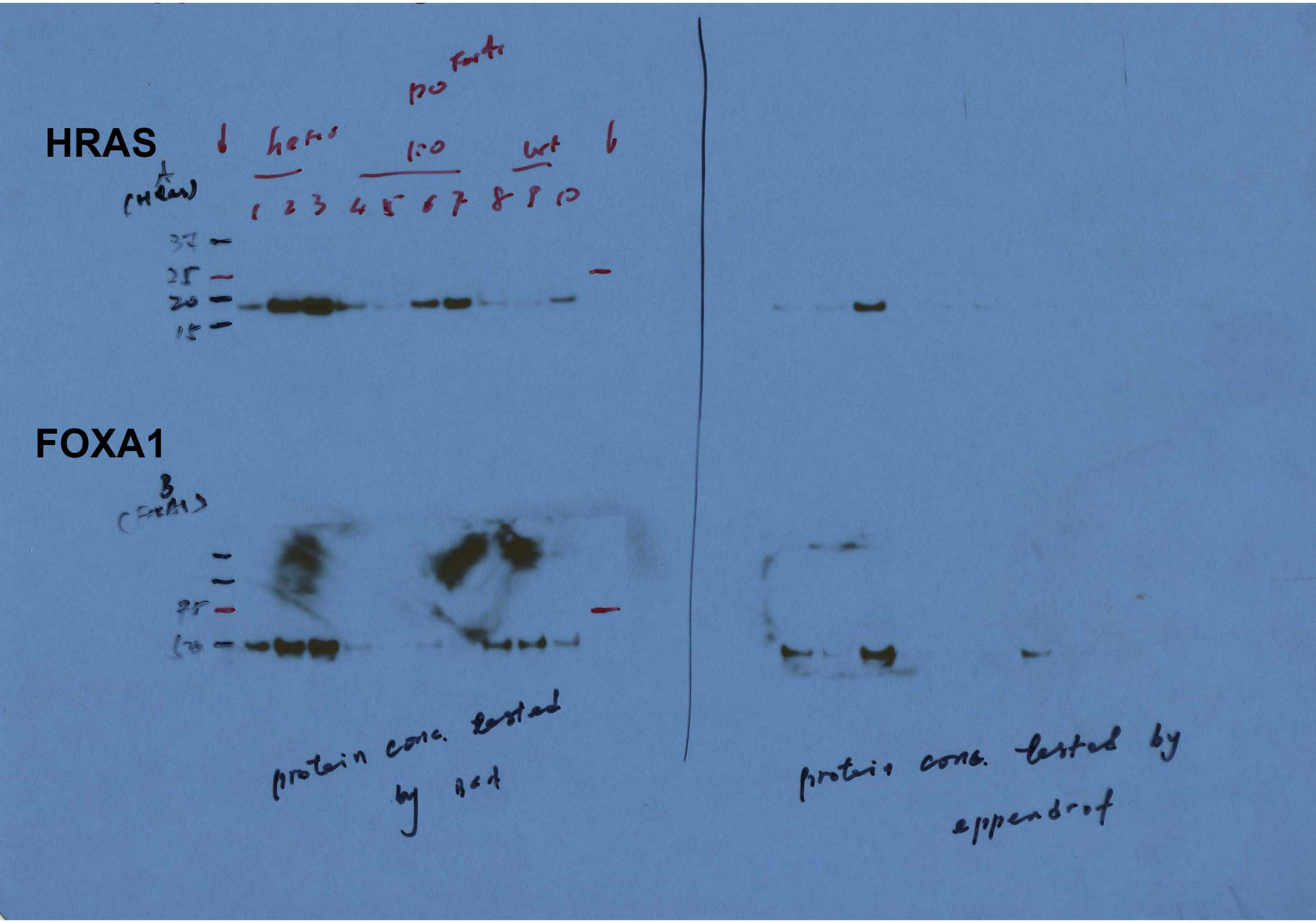

Supplemental Figure 3

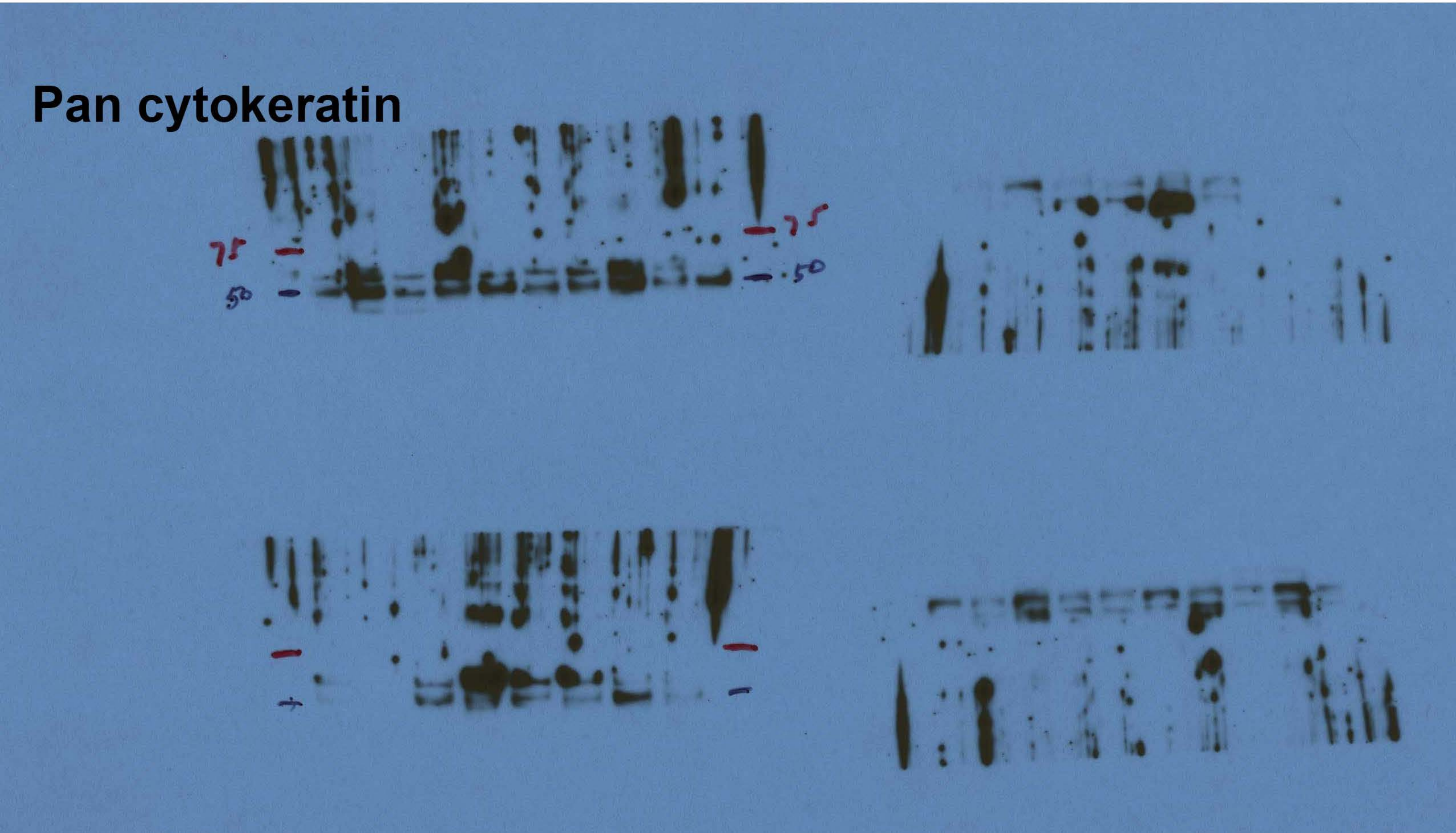

Supplemental Figure 4

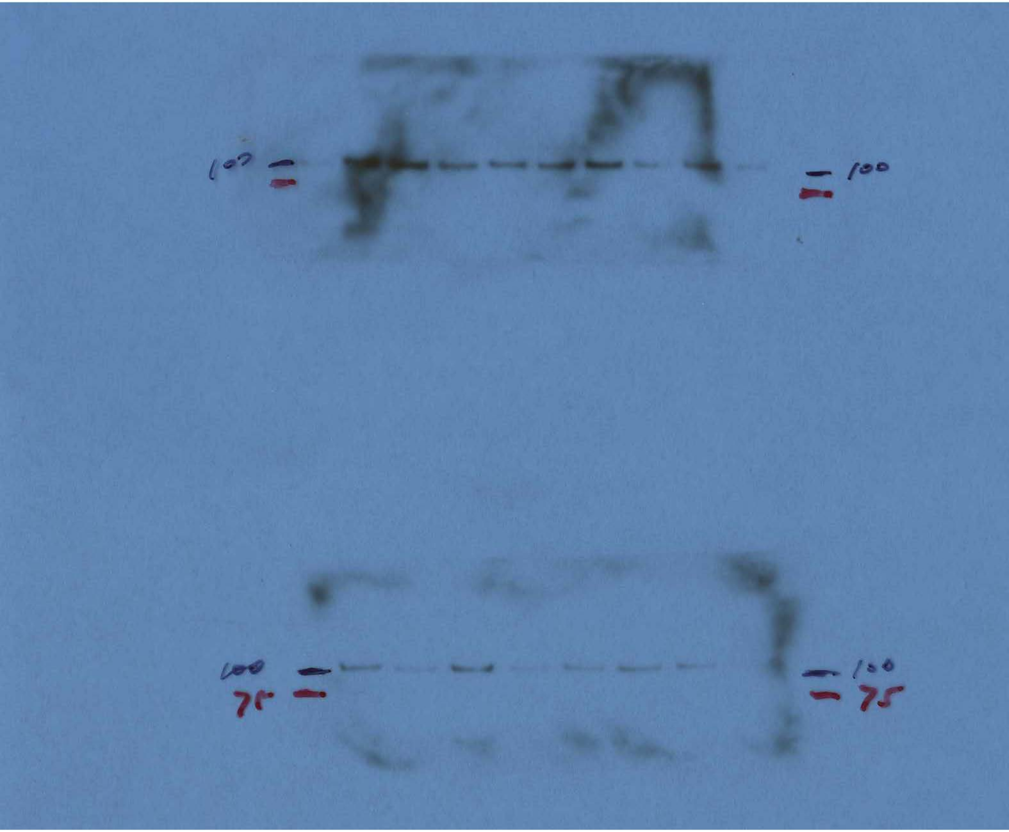

Supplement: Supplementary file 1 — Supplemental data [file 41598_2018_36720_MOESM1_ESM.pdf]
